# Elevated human placental heat shock protein 5 is associated with spontaneous preterm birth

**DOI:** 10.1038/s41390-023-02501-9

**Published:** 2023-02-14

**Authors:** Pinja Tissarinen, Heli Tiensuu, Antti M. Haapalainen, Tomi A. Määttä, Marja Ojaniemi, Mikko Hallman, Mika Rämet

**Affiliations:** 1 PEDEGO Research Unit and Medical Research Center Oulu, University of Oulu, Oulu, Finland; 2 Department of Children and Adolescents, Oulu University Hospital, Oulu, Finland; 3 Faculty of Medicine and Health Technology, Tampere University, Tampere, Finland

## Abstract

**Background:**

Specific heat shock proteins are associated with pregnancy complications, including spontaneous preterm birth (SPTB). Placental proteomics and whole exome sequencing recently suggested an association between heat shock protein HSPA5 and uncomplicated SPTB. In the present study, we investigated the localization of and possible roles for HSPA5 in SPTB.

**Methods:**

Western blot was performed to validate the result from the previously published proteomic analysis. We used qPCR to assess mRNA expression of genes and immunohistochemistry and immunoelectron microscopy to examine localization of HSPA5 in placental tissue. We silenced the *HSPA5* gene in the HTR8/SVneo human trophoblast cell line to investigate possible functions of HSPA5.

**Results:**

HSPA5 was upregulated in placentas from SPTBs compared to spontaneous term births. We did not observe upregulation of *HSPA5* mRNA in placental samples. The protein was localized in placental trophoblast in both spontaneous preterm and term placentas. Gene silencing of *HSPA5* in human trophoblast cell culture affected the inflammatory response and decreased the expression of several proinflammatory genes.

**Conclusions:**

We suggest that upregulation of HSPA5 in the placenta is associated with spontaneous preterm labor. HSPA5 may promote the inflammatory response and alter the anti-inflammatory state of the placenta which could eventually lead to premature labor.

**Impact:**

- We validated upregulation of HSPA5 in placentas from spontaneous preterm birth. *HSPA5* was not upregulated at transcriptional level which suggests that it may be regulated post-translationally.
- Silencing *HSPA5* in a human trophoblast–derived cell line suggested that HSPA5 promotes expression of proinflammatory cytokines. The emerging inflammation could lead to spontaneous preterm labor.
- Identifying inflammatory pathways and factors associated with spontaneous preterm birth increases knowledge of the molecular mechanisms of premature labor. This could provide cues to predict imminent premature labor and lead to information about how to safely maintain pregnancies.

## Introduction

Preterm birth (PTB) is defined as live birth before 37 completed weeks of pregnancy; it is a leading cause of death among children under 5 years of age.^1–4^ Known risk factors for PTB include, for example, previous history of PTBs, multiple pregnancy, and infection.^2,5–7^ Approximately 40–50% of spontaneous preterm births occur without a known complication.^5,6,8^ Spontaneous preterm and term labor may share molecular pathways,^5,9^ but the mechanisms that initiate birth are poorly understood.^4,10,11^

Heat shock proteins (HSPs) are associated with adverse pregnancy outcomes, such as preeclampsia, premature rupture of fetal membranes, and spontaneous preterm birth (SPTB).^12–14^ HSPs are expressed in all tissues, and their expression profile is mostly low under nonstressed physiological conditions.^15–17^ For example, HSPs are present in the human placenta,^18,19^ human reproductive tissues,^16^ and mammalian embryos.^20^ Some HSPs are constitutively expressed, while others are induced in response to a stimulus.^21,22^ HSP levels increase under adverse conditions to protect the cell from damage.^15,23^ The function of HSPs depends on their localization. Intracellular HSPs mainly protect the cell from damage and participate in protein folding and transporting proteins; they also prevent the aggregation of misfolded proteins.^14,23–25^ Extracellular HSPs have immunological functions.^14,24^ Some HSPs are known to stimulate innate and adaptive immune systems^23^ and induce proinflammatory cytokine production^26^ via toll like receptors.^17,27^

HSPs can be categorized into subfamilies according to their molecular weight (kDa): small HSPs, HSP40, HSP60, HSP70, HSP90, and HSP110.^23,26,28^ The HSP70 family (also known as the HSPA family) is one of the most studied HSP families^21,29–31^ and includes 13 members.^27,30^ The members have different locations and functions in the cell. For example, HSPA9 is a mitochondrial housekeeping protein and HSPA5 is localized mainly in the endoplasmic reticulum.^30^ HSPA1A and HSPA8 participate in antigen processing and presentation^29^ and may promote changes in placental immune tolerance.^32^

The current understanding of initiation of spontaneous labor at term involves a gradual shift of the uterus and placenta from an anti-inflammatory to a proinflammatory state.^9,10,33^ This immunomodulatory switch promotes formation of labor-producing mediators and may alter fetomaternal tolerance.^33,34^ The transition may be initiated by a change in the signaling of inflammatory pathways, such as changes in chemokine and cytokine levels.^10,33^ In SPTB, the shift in the immunological state of the uterus and placenta may activate too early.^33,35^ Previous studies have proposed that HSPs may disturb fetomaternal tolerance at the onset of labor.^12–14,18^ For example, HSP70 antigen–antibody complexes have been observed in SPTB placentas.^18^ Moreover, the circulating HSP60/HSP70 ratio is higher in women who have spontaneous miscarriage.^36^ Huusko et al. (2021) proposed that to maintain pregnancy, HSP production is suppressed.^12^

In a previous investigation, we characterized the proteomes of placentas from SPTBs, medically-indicated PTBs and spontaneous term births.^37^ We discovered six SPTB-associated placental proteins in the proteomics data; HSPA5 was one of them. According to the proteomics and western blot, HSPA5 was significantly upregulated in placentas from SPTBs. Additionally, a variant of *HSPA5* may predispose to SPTB due to a probably harmful amino acid change.^12,37^ As some HSPs have been associated with SPTB previously,^12,13^ we investigated the possible functions of HSPA5 in predisposition to SPTB. We discovered that HSPA5 has a role in promoting the expression of proinflammatory cytokines in premature labor.

## Materials and methods

### Ethical statement

Written informed consent was obtained from all participants or their guardian(s) and the present study was approved by the Northern Ostrobothnia Hospital District Ethical Committee (79/2003 and 73/2013; amendments).

### Collection of placental samples

In short, placental samples from basal plate (maternal side of the placenta) were collected at Oulu University Hospital in 2010–2016, as described previously.^38^ The inclusion criterion for gestational age (GA) was <37 weeks for SPTB and >38 weeks for STB samples. Clinical characteristics of the pregnancies are presented in Table 1. In total, 22 placental samples from SPTBs and 23 placental samples from STBs were collected. None of the deliveries was provider-initiated. Diagnosis of chorioamnionitis was based on clinical findings. One SPTB sample was from a twin pregnancy, otherwise the samples were from singleton pregnancies.

**Table 1:** Clinical characteristics of the pregnancies.

Parameter	SPTB *n* = 22	STB *n* = 23	*p* value^a^
GA			
Median (days)	223.5	283.0	<0.001
Median (weeks)	31.9	40.4	<0.001
Birth weight, median (g)	2085.0	3870.0^b^	<0.001
Delivery type			
Vaginal delivery (%)	14 (63.6)	23 (100.0)	N/A
Cesarean section (%)	8 (36.4)	0 (0.0)	N/A
Chorioamnionitis, clinically			
Yes (%)	5 (22.7)	0 (0.0)	N/A
No (%)	9 (40.9)	14 (60.9)	N/A
N/A (%)	8 (36.4)	9 (39.1)	N/A
Preeclampsia			
Yes (%)	0 (0.0)	0 (0.0)	N/A
No (%)	20 (90.9)	21 (91.3)	N/A
N/A (%)	2 (9.1)	2 (8.7)	N/A
Pregnancy type			
Singleton (%)	21 (95.5)	23 (100.0)	N/A
Twin (%)	1 (4.5)	0 (0.0)	N/A

SPTB spontaneous preterm birth, STB spontaneous term birth, GA gestational age, N/A not available or not applicable.

Placental samples were collected at Oulu University Hospital during 2010–2016 immediately after delivery. The samples were collected from the maternal side of the placenta.

^a^Mann–Whitney *U* test was used to test difference between SPTB and STB groups.

^b^Three cases from STBs were missing birth weight.

### Western blotting of HSPA5

A quantitative western blot method was used to validate the proteomic finding of HSPA5 (P11021) as described previously.^37,39^ All samples (SPTB *n* = 10 [GA from 26 weeks + 1 day to 35 weeks + 2 days], STB *n* = 14 [GA from 38 weeks + 6 days to 41 weeks + 5 days]) were normalized against the reference protein tubulin α-1B. We used mouse monoclonal anti-human HSPA5 antibody (MAB4846, 1:1000 dilution; R&D Systems, Minneapolis, Minnesota) and rabbit monoclonal anti-human tubulin α-1B antibody (NB110-57609, 1:5000 dilution; Novusbio, Abingdon, United Kingdom) to detect HSPA5 and tubulin α-1B, respectively (Supplementary Fig. S1). Normalized protein expression ratios were used in the analysis.

### qPCR of placental *HSPA5*

We performed qPCR to determine mRNA levels of *HSPA5* in placental samples from the basal plate of the placenta. We compared samples from SPTBs (*n* = 18 [GA from 25 weeks to 36 weeks + 6 days]) and STBs (*n* = 23 [GA from 39 weeks to 41 weeks + 6 days]). Placental RNA was isolated as described previously.^38^

Isolated RNA was converted into cDNA with the Transcriptor First Strand cDNA Synthesis Kit (Roche Diagnostics, Risch-Rotkreuz, Switzerland) according to the standard procedure. cDNA samples were diluted 1:2 with RNase-free H_2_O. A LightCycler96 instrument (Roche Diagnostics) was used to assess relative quantification of *HSPA5*. Cytochrome C1 (*CYC1*) was used as a reference gene to normalize the measured mRNA levels. Relative quantification was determined with the ΔΔ cycle threshold method.^38^ Primers and probes are listed in Supplementary Table S1. Each qPCR measurement was done in triplicate.

### Immunohistochemical staining of HSPA5

To visualize the location of HSPA5, SPTB (*n* = 6) and STB (*n* = 6) placental samples were analyzed with immunohistochemical staining. The immunohistochemical staining procedure was previously described in detail.^40^ Samples were incubated with rabbit anti-human HSPA5 antibody (3177, 1:4000 dilution; Cell Signaling Technology, Danvers, Massachusetts) for detection. Non-immune rabbit IgG was used for negative controls.

### Immunoelectron microscopy of HSPA5

Immunoelectron microscopy (immuno-EM) was performed at the Biocenter Oulu Electron Microscopy Core Facility as described previously.^37^ In short, fresh human placental samples from SPTBs and STBs were fixed and cut with a Leica EM UC7 cryoultramicrotome (Leica Microsystems, Vienna, Austria). For immunolabeling, sections of Butvar-coated nickel grids were exposed to primary antibody to HSPA5 (3177, 1:100 dilution; Cell Signaling Technology) and bound antibodies were labeled by incubation with protein A–conjugated 10 nm gold (Cell Microscopy Core, University Medical Center Utrecht, The Netherlands). Controls were prepared by replacing the primary antibody with PBS. To reduce background labeling, endogenous immunoglobulins were blocked using Fab fragments (Goat Anti-Human IgG [H + L]; Jackson ImmunoResearch Europe Ltd, United Kingdom). Samples were incubated with Fab fragments for 30 min after primary blocking step before incubation with primary antibody. Thin sections were examined with a Tecnai G2 Spirit 120 kV transmission electron microscope (FEI, Eindhoven, The Netherlands), and images were captured by a Quemesa CCD camera (Olympus Soft Imaging Solutions GMBH, Münster, Germany).

### Gene knockdown of *HSPA5* with small interfering RNAs (siRNAs) in HTR8/SVneo human placental cell line

Gene silencing of *HSPA5* was performed in the human placental trophoblast cell line HTR8/SVneo (CRL-3271™; ATCC, Manassas, Virginia). The procedure for gene silencing was described in detail previously.^40^ Cells were grown in RPMI-1640 growth medium (Thermo Fisher Scientific, Waltham, Massachusetts). The growth medium was supplemented with 10% fetal bovine serum (FBS; Sigma-Aldrich, St. Louis, Missouri) and 1× penicillin/streptomycin (Sigma-Aldrich). Cells were grown at 37 °C (5% CO_2_, humidified atmosphere), and 0.05% trypsin/0.02% EDTA was used in subculturing. HTR8/SVneo cells were reverse and forward transfected with siRNAs targeting *HSPA5* (sense GAUAAUCAACCAACUGUUA, antisense UAACAGUUGGUUGAUUAUC) (Sigma-Aldrich). For the control, MISSION siRNA Universal Negative Control #1 (Sigma-Aldrich) was transfected in the same way as the siRNAs that targeted *HSPA5*. Lipofectamine 3000 (Invitrogen, Carlsbad, California) was used as a transfection reagent. Cells (100,000/well) were incubated with 10 nM siRNA concentration in the reverse transfection. Forward transfection was performed after 24 h of incubation. In the forward transfection, cells were transfected again with siRNA concentrations of 10 nM. Cells were incubated with siRNAs for 48 h and then harvested with 1× trypsin–EDTA (Sigma-Aldrich).

### Transcriptomic analysis of *HSPA5*-silenced HTR8/SVneo cells

For transcriptomic analysis of *HSPA5*-silenced cells and cells treated with siRNA Universal Negative Control #1, cells were disrupted with a 20 G needle and 1 ml syringe. RNA isolation was done with the RNeasy Micro Kit (Qiagen, Venlo, the Netherlands). The quality of isolated RNA was checked with an Agilent 2100 Bioanalyzer system at the Biocenter Oulu Sequencing Center, Finland. RNA sequencing was done at the Finnish Functional Genomics Center (FFGC; Turku, Finland). Transcriptomes of HSPA5-silenced cells (*n* = 3) and negative control-cells (*n* = 3) were determined with the Illumina HiSeq high‐throughput sequencing system. Sequencing data were analyzed by the Bioinformatics Unit Core Service at the Turku Center for Biotechnology, Finland.

### Verification of selected genes in *HSPA5*-silenced cells using qPCR

Gene silencing of *HSPA5* in the silenced cells was verified by qPCR with a larger number of specimens (*HSPA5*-silenced cells *n* = 6, negative control cells *n* = 6). Isolated RNA from HTR8/SVneo cells was converted into cDNA as described earlier. *AP2A1*, *TNFRSF9*, *HSP90B1*, *CXCL8* and *CCL2* were chosen from the RNA sequencing data of the *HSPA5* silencing experiment for verification. qPCR was performed as described in detail in the qPCR of placental *HSPA5* section above. Primers and probes are listed in Supplementary Table S1.

### Statistical analysis

SPSS Statistics 26.0 (IBM Corporation, Armonk, New York) was used to assess statistical significance. Nonparametric Mann–Whitney *U* test was applied to evaluate differences between SPTB and STB groups. Student’s *t* test was applied to discover difference in RNA sequencing. Differences were considered statistically significant when the test resulted in a *p* value of <0.05.

## Results

### HSPA5 is upregulated in placentas from SPTBs

**Fig. 1:**
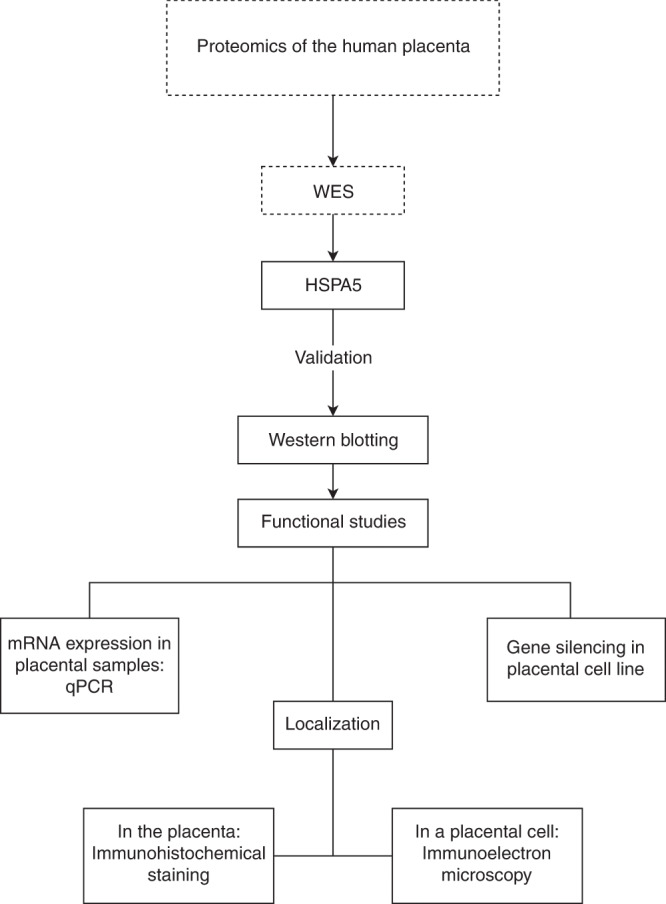
Flow chart of the present study. We previously used proteomics^37^ to identify six proteins, including HSPA5, with expression levels associated with SPTB. We then used WES to identify potentially damaging variants in families with recurrent SPTBs.^13,37^ In this study, we validated the proteomic result, and investigated the function of HSPA5 in SPTB by immunohistochemistry, immunoelectron microscopy, and siRNA-mediated gene silencing.

In a previous investigation, we discovered that HSPA5 was upregulated in the placentas from SPTBs.^37^ We therefore set out to characterize the function and localization of HSPA5 in the placenta (Fig. 1).

**Fig. 2:**
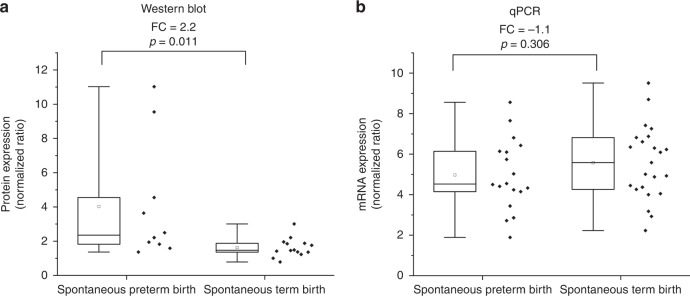
Placental protein expression of HSPA and mRNA expression of *HSPA5* in SPTBs and STBs. Placental samples were from the basal plate of the placenta. Protein expression was used to validate the proteomic finding of HSPA5 as described previously.^37^ Protein levels normalized against the reference protein tubulin α-1B (**a**). Relative mRNA expression of *HSPA5* assessed by qPCR. mRNA levels normalized against the housekeeping gene *CYC1* (**b**). Statistical analysis was performed with Mann–Whitney *U* test to discover differences. Expression ratio (fold change [FC]) between compared groups presented in the figures. Quartiles displayed by a box and whiskers. Ends of whiskers represent minimum and maximum values, excluding outliers. Inside box, median is indicated with a line and mean value is represented as a square.

First, we evaluated the placental protein level of HSPA5 by western blot (Supplementary Fig. S1). HSPA5 protein expression was upregulated by 2.2-fold in the basal plate in SPTB placentas (*p* = 0.011, Fig. 2a).

To determine whether *HSPA5* is upregulated at the transcriptional level, we analyzed mRNA expression of *HSPA5* in placental samples from SPTBs and STBs by qPCR. We did not observe a statistically significant difference in *HSPA5* mRNA expression between SPTB and STB placentas (*p* = 0.306, Fig. 2b). These results suggest that HSPA5 is not regulated at the mRNA level; rather, it is post-translationally regulated. Next, we studied whether a certain variant of the corresponding gene, *HSPA5*, affects the function of HSPA5 protein as the recently published WES data suggested an association between the variant and recurrent preterm birth.^12,37^

### SPTB-associated variant of *HSPA5* may cause changes in the physiochemical properties of HSPA5

We recently investigated the presence of rare (MAF < 1%) and common (MAF < 10%) potentially damaging variants in the *HSPA5* gene by using WES data.^12,13,37^ The WES data comprised Finnish and Danish mothers who gave birth preterm and Finnish infants who were born preterm. One variant of *HSPA5* (rs56136100) was found in two unrelated families.^12,37^ *HSPA5* variant rs56136100 was shared by two mothers who gave birth preterm.^12^ rs56136100 is a non-conservative missense variant (E557G), and it changes glutamic acid to glycine.^12^ Due to the change from an acidic to a hydrophobic amino acid, the variant may affect the physiochemical properties of HSPA5.^12^ Moreover, the variant is predicted to be damaging by several in silico tools and has a Combined Annotation Dependent Depletion (CADD) score of 33.^12^

**Fig. 3:**
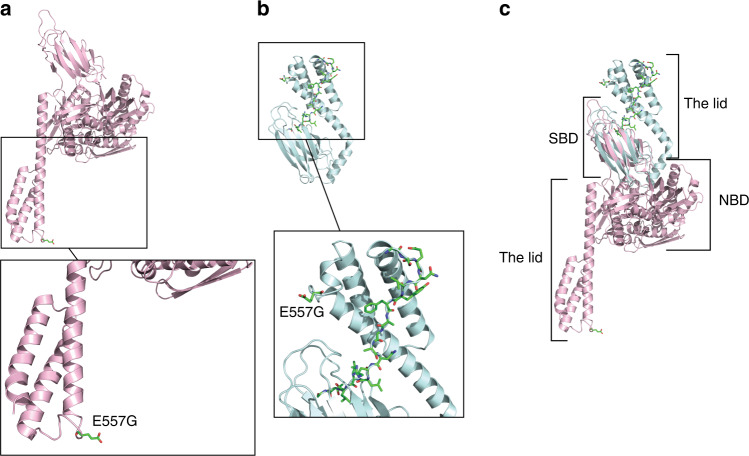
Crystal structure of human HSPA5. HSPA5 has a nucleotide-binding domain (NBD) and a substrate-binding domain (SBD).^42,44^ **a** Crystal structure of HSPA5 when ATP is bound in the NBD (PDB code 5E84.^42^) SBD is in open conformation. **b** Crystal structure of HSPA5 with SBD in closed conformation (PDB code 5E85.^42^) This crystal structure lacks the NBD. C-terminal alpha helices, also known as the lid, cover the substrate in the binding pocket.^25,42,44^ Substrate (peptide substrate for DnaK [NR peptide]) in SBD is shown in stick representation, where oxygen, nitrogen, and carbon are colored in red, blue, and green, respectively. **c** Superposition of open and closed conformations shown in (**a**) and (**b**) using SBD only. Structures (**a**–**c**) are drawn as ribbons, and the site of the amino acid change (E557G) is displayed as a stick drawing. Open and the closed conformations are colored in pink and cyan, respectively. Crystal structures were illustrated with PyMOL.

We made an illustration of the crystal structure of HSPA5 with the program PyMOL to visualize the location of the affected amino acid (E557G) and to examine whether it could affect the function of the protein (Fig. 3). HSPA5 has two domain structures that are conserved in all members of the HSP70 family: an N-terminal ATPase domain (nucleotide-binding domain, NBD) and a C-terminal substrate-binding domain (SBD).^25,41–43^ The SBD consists of two subdomains: a binding pocket for the substrate and a helical lid (alpha helices) to cover the binding pocket.^25,42,44^ The amino acid sequence for the alpha helical lid is from 538 to 607.^45^ Thus, the site of the amino acid change (E557G) is in the SBD, as illustrated in Fig. 3. When ATP is bound to the NBD, the affinity of the SBD for a substrate is drastically reduced^44^ and consequently the alpha helical lid of the SBD is in an open conformation, as presented in Fig. 3a. When ATP is hydrolyzed to ADP, a substrate can bind to the SBD,^44^ which changes the structure from an open to a closed conformation (Fig. 3b). To determine how these two conformations are positioned relative to one another, we aligned the two conformations (Fig. 3c). As seen in Fig. 3c, the C-terminal alpha helical lid closes the substrate in the binding pocket.^25,42,44^ In the open and closed conformations, E557 is solvent exposed and does not have hydrogen bonds with neighboring amino acids. This suggests that the E557G change does not affect the structure of HSPA5.

### HSPA5 is localized in placental trophoblasts

**Fig. 4:**
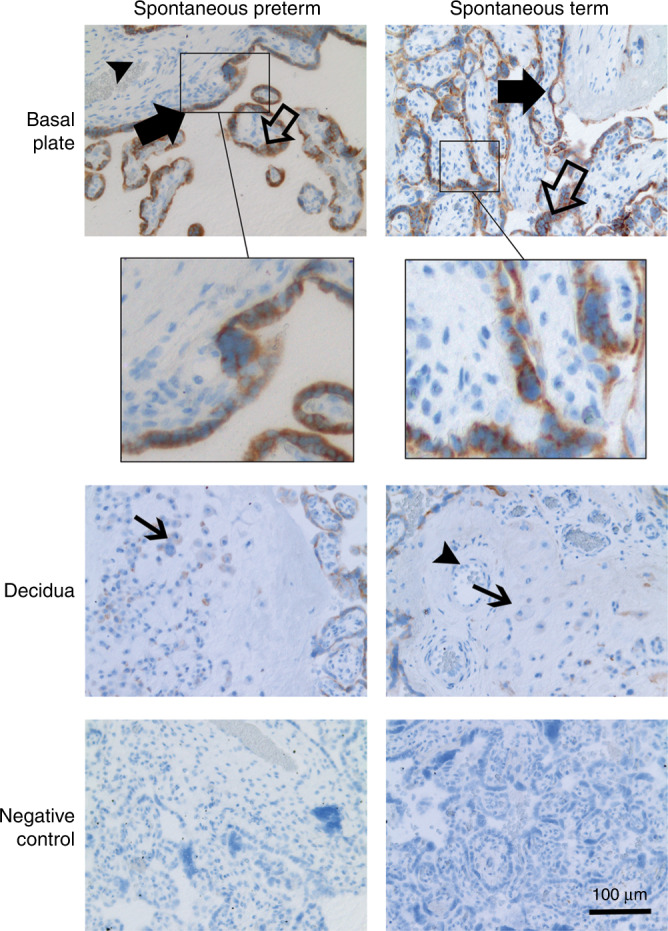
Placental localization of HSPA5 in spontaneous preterm and term births. Localization of HSPA5 in SPTB and STB placentas. In total, 12 placentas (SPTBs *n* = 6, STBs *n* = 6) were immunostained with anti‐human HSPA5 antibody. Samples were from the basal plate (maternal side) of the placenta. Immunostaining is indicated by filled large arrows in cytotrophoblast, unfilled large arrows in syncytiotrophoblast, filled small arrows in decidual trophoblast, and filled arrowheads in capillary endothelial cells. Original magnification is ×20 in all figures. Control represents the isotype controls for immunostaining. Scale bar represents 100 μm.

To determine the location of HSPA5 in placental tissue, we performed immunohistochemical staining of HSPA5 in placental tissue samples from SPTBs and STBs. The samples were from the basal plate of the placenta. We did not observe differences in the staining between SPTB and STB groups. Staining was strong for HSPA5 in cytotrophoblast and syncytiotrophoblast in the basal plate. In the decidua, staining was evident in decidual trophoblast. Staining was low or nonexistent in capillary endothelial cells. Immunostaining of SPTB and STB placenta samples for HSPA5 is shown in Fig. 4.

To characterize the subcellular localization of HSPA5 in the placenta, we used immuno-EM. Samples were from SPTBs and STBs, and from the basal plate of the placenta. HSPA5 was present in placental trophoblasts, mainly in the cytoplasm (Supplementary Fig. S2a and c). We did not detect HSPA5 in syncytial microvilli. We did not detect clear difference in the localization of HSPA5 between SPTB and STB placentas in immuno-EM.

### siRNA-induced gene silencing of *HSPA5* causes changes in expression levels of inflammation-related factors

We silenced *HSPA5* expression in the HTR8/SVneo human trophoblast-derived cell line with small interfering RNAs (siRNAs) to study the function of *HSPA5* in the placenta. Silencing percentages were 54% (qPCR) and 45% (RNA sequencing), respectively (Supplementary Fig. S3). We characterized the transcriptomes of HSPA5-silenced cells and cells treated with siRNA Universal Negative Control #1. In the analysis, the thresholds used were a false discovery rate (FDR)–adjusted *p* value of ≤0.05 and a fold change (FC) of ≥1.5. The transcriptional analysis revealed 43 upregulated (Supplementary Table S2) and 50 downregulated (Supplementary Table S3) genes after *HSPA5* silencing.

The IL-17 signaling pathway was the most affected pathway in the KEGG pathway database search (Table 2). Gene silencing of *HSPA5* affected eight genes in this pathway, including several chemokines such as *CCL2* and *CXCL8* (Table 3). According to a Gene Ontology (GO) search, the top biological pathways (GO Biological Process [GO-BP]) affected by *HSPA5* silencing were inflammatory response, response to toxic substance, and angiogenesis (Table 4). Additionally, *HSPA5* silencing tended to affect genes that promote extracellular matrix organization and extracellular space (GO Cellular Component search [GO-CC]) (Supplemental Table S4).

**Table 2:** KEGG pathway database search of genes affected by *HSPA5* silencing.

Term	Count	%	*p* value^a^	Benjamini
IL-17 signaling pathway	8	8.7	2.00E–06	3.70E–04
Rheumatoid arthritis	7	7.6	2.60E–05	2.40E–03
Pertussis	6	6.5	1.20E–04	7.40E–03
TNF signaling pathway	6	6.5	7.10E–04	3.40E–02
Bladder cancer	4	4.3	2.20E–03	7.00E–02
Lipid and atherosclerosis	7	7.6	2.40E–03	7.00E–02
Cytokine-cytokine receptor interaction	8	8.7	2.60E–03	7.00E–02
NF-kappa B signaling pathway	5	5.4	4.30E–03	1.00E–01
Pathways in cancer	10	10.9	6.00E–03	1.30E–01
PI3K-Akt signaling pathway	8	8.7	7.00E–03	1.30E–01
Kaposi sarcoma-associated herpesvirus infection	6	6.5	7.80E–03	1.30E–01
MAPK signaling pathway	7	7.6	1.10E–02	1.70E–01
Fluid shear stress and atherosclerosis	5	5.4	1.20E–02	1.70E–01
Coronavirus disease - COVID-19	6	6.5	1.60E–02	2.20E–01
Salmonella infection	6	6.5	2.10E–02	2.70E–01
Staphylococcus aureus infection	4	4.3	2.30E–02	2.70E–01
Prostate cancer	4	4.3	2.40E–02	2.70E–01
Viral protein interaction with cytokine and cytokine receptor	4	4.3	2.60E–02	2.70E–01
Chagas disease	4	4.3	2.70E–02	2.70E–01
NOD-like receptor signaling pathway	5	5.4	3.00E–02	2.80E–01
Transcriptional misregulation in cancer	5	5.4	3.40E–02	3.10E–01
Focal adhesion	5	5.4	3.90E–02	3.40E–01
Chemical carcinogenesis—receptor activation	5	5.4	4.60E–02	3.80E–01

^a^KEGG pathways with *p* < 0.05 are shown.

*HSPA5* silenced in HTR8/SVneo commercial cell line by siRNA. Transcriptome of these cells compared with transcriptome of cells treated with negative siRNA. KEGG pathway database terms ranked based on *p* value. Threshold of *p* value was <0.05.

**Table 3:** Genes related to IL-17 signaling pathway after *HSPA5* silencing.

Gene name	*p* value^a^	adj. *p* value^b^	FC^c^
C-X-C motif chemokine ligand 8 (*CXCL8*)	0.00020	0.0000	–1.92
C-C motif chemokine ligand 2 (*CCL2*)	0.00011	0.0000	–1.91
C-X-C motif chemokine ligand 6 (*CXCL6*)	0.00016	0.0000	–1.89
C-X-C motif chemokine ligand 1 (*CXCL1*)	0.00022	0.0000	–1.89
Heat shock protein 90 beta family member 1 (*HSP90B1*)	0.0001	0.0000	1.85
Matrix metallopeptidase 1 (*MMP1*)	0.0023	0.0156	1.81
Fos proto-oncogene, AP-1 transcription factor subunit (*FOS*)	0.0006	0.0066	1.56
TNF alpha induced protein 3 (*TNFAIP3*)	0.00054	0.0000	–1.51

^a^*t* test *p* value between sample groups (*HSPA5*-silenced and negative-control cells).

^b^False discovery rate (FDR)–adjusted *p* value.

^c^Expression ratio (FC) between compared sample groups. Comparison between *HSPA5-*silenced cells and negative control cells.

KEGG pathway database search revealed that after the silencing of *HSPA5*, IL-17 signaling pathway had the lowest *p* value of the affected KEGG pathways. Silencing of *HSPA5* affected eight of the 93 genes in this pathway. Terms ranked by FC.

**Table 4:** GO gene ontology biological process (GO-BP) search of genes affected by *HSPA5*-silencing.

Term	Count	%	*p* value^a^	Benjamini
Inflammatory response	14	15.6	3.00E–08	2.50E–05
Response to toxic substance	6	6.7	5.60E–05	2.30E–02
Angiogenesis	8	8.9	1.00E–04	2.80E–02
Extracellular matrix organization	7	7.8	3.70E–04	7.50E–02
Signal transduction	16	17.8	3.90E–04	6.40E–02
Positive regulation of apoptotic cell clearance	3	3.3	4.80E–04	6.50E–02
Negative regulation of cell proliferation	9	10	6.40E–04	7.40E–02
Blood vessel development	4	4.4	8.10E–04	8.20E–02
PERK-mediated unfolded protein response	3	3.3	1.50E–03	1.30E–01
Skeletal muscle cell differentiation	4	4.4	1.70E–03	1.30E–01
Chemotaxis	5	5.6	2.90E–03	2.00E–01
Cell chemotaxis	4	4.4	3.80E–03	2.40E–01
Immune response	8	8.9	4.20E–03	2.40E–01
Positive regulation of endothelial cell proliferation	4	4.4	4.50E–03	2.40E–01
Chemokine-mediated signaling pathway	4	4.4	4.90E–03	2.40E–01
Cell adhesion	8	8.9	6.70E–03	3.00E–01
Positive regulation of vascular endothelial growth factor production	3	3.3	7.50E–03	3.10E–01
Regulation of complement activation	3	3.3	9.20E–03	3.50E–01
Cellular response to fibroblast growth factor stimulus	3	3.3	9.20E–03	3.50E–01
Transforming growth factor beta receptor signaling pathway	4	4.4	1.00E–02	3.60E–01
Positive regulation of angiogenesis	4	4.4	1.80E–02	5.40E–01
Regulation of cell adhesion	3	3.3	1.80E–02	5.20E–01
Response to peptide hormone	3	3.3	1.90E–02	5.20E–01
Positive regulation of glomerular filtration	2	2.2	1.90E–02	5.10E–01
Apoptotic process	8	8.9	2.00E–02	5.00E–01
Negative regulation of endopeptidase activity	4	4.4	2.10E–02	5.10E–01
Positive regulation of fibroblast proliferation	3	3.3	2.80E–02	6.00E–01
Mammary gland involution	2	2.2	2.90E–02	6.00E–01
Protein ubiquitination	6	6.7	3.00E–02	6.00E–01
Cellular response to hydrogen peroxide	3	3.3	3.10E–02	6.00E–01
Cellular response to organic cyclic compound	3	3.3	3.30E–02	6.10E–01
Cell-cell signaling	5	5.6	3.40E–02	6.10E–01
DNA damage response, signal transduction by p53 class mediator resulting in cell cycle arrest	3	3.3	3.60E–02	6.20E–01
Positive regulation of gene expression	5	5.6	3.80E–02	6.30E–01
Blood vessel maturation	2	2.2	3.80E–02	6.20E–01
Cellular response to antibiotic	2	2.2	3.80E–02	6.20E–01
Positive regulation of protein ubiquitination	3	3.3	3.80E–02	6.10E–01
Positive regulation of vascular associated smooth muscle cell migration	2	2.2	4.30E–02	6.40E–01
Response to molecule of bacterial origin	2	2.2	4.30E–02	6.40E–01
ATF6-mediated unfolded protein response	2	2.2	4.30E–02	6.40E–01
Negative regulation of platelet aggregation	2	2.2	4.30E–02	6.40E–01
Positive regulation of I-kappab kinase/NF-kappab signaling	4	4.4	4.30E–02	6.30E–01
Response to lipopolysaccharide	4	4.4	4.50E–02	6.40E–01
Intracellular signal transduction	6	6.7	4.50E–02	6.30E–01

^a^Gene ontologies with *p* < 0.05 are shown.

*HSPA5* silenced in HTR8/SVneo commercial cell line by siRNA. Transcriptome of these cells compared with transcriptome of cells treated with negative siRNA. GO-BP terms ranked based on *p* value. Threshold of *p* value was <0.05.

Of the affected biological pathways (GO-BP), inflammatory response had the lowest *p* value (*p* = 2.5E–5) (Table 4). In this gene ontology, 14 of the 93 genes were affected by *HSPA5* silencing (Table 5); most of these 14 genes were downregulated. The affected genes included proinflammatory cytokines and chemokines, such as *CCL2*, *IL1A*, and *CXCL8* (Table 5). CCL2 is an important regulator of monocyte migration and activation.^35^ Additionally, expression of interleukin 1A (a member of the interleukin 1 family) increases in response to inflammatory stimuli^46^ and can stimulate myometrial contractions.^47^ Interleukin 8 (IL-8), encoded by *CXCL8*, is a chemoattractant;^48^ elevated levels of *CXCL8* and IL-8 have been observed during labor.^49^ Overall, these data suggest that HSPA5 has immunomodulatory functions in the placenta.

**Table 5:** Genes related to inflammatory response after *HSPA5* silencing.

Gene name	*p* value^a^	adj. *p* value^b^	FC^c^
Complement C3 (*C3*)	0.00005	0.0000	–2.26
TNF receptor superfamily member 9 (*TNFRSF9*)	0.00003	0.0000	–2.21
C-X-C motif chemokine ligand 8 (*CXCL8*)	0.00020	0.0000	–1.92
C-C motif chemokine ligand 2 (*CCL2*)	0.00011	0.0000	–1.91
C-X-C motif chemokine ligand 6 (*CXCL6*)	0.00016	0.0000	–1.89
C-X-C motif chemokine ligand 1 (*CXCL1*)	0.00022	0.0000	–1.89
Phospholipase A2 group IVC (*PLA2G4C*)	0.00008	0.0000	–1.85
Secretogranin II (*SCG2*)	0.00038	0.0000	–1.72
Interleukin 1 alpha (*IL1A*)	0.00061	0.0066	–1.65
Complement C4A (Rodgers blood group) (*C4A*)	0.00067	0.0066	–1.57
E74 like ETS transcription factor 3 (*ELF3*)	0.00088	0.0066	–1.57
Fos proto-oncogene, AP-1 transcription factor subunit (*FOS*)	0.0006	0.0066	1.56
RELB proto-oncogene, NF-kb subunit (*RELB*)	0.00070	0.0066	–1.52
TNF alpha induced protein 3 (*TNFAIP3*)	0.00054	0.0000	–1.51

^a^*t* test *p* value between sample groups (*HSPA5*-silenced and negative-control cells).

^b^False discovery rate (FDR)–adjusted *p* value.

^c^Expression ratio (FC) between compared sample groups. Comparison between *HSPA5-*silenced cells and negative control cells.

GO-BP search revealed that after the silencing of *HSPA5*, inflammatory response had the lowest *p* value of the affected biological pathways. Silencing of *HSPA5* affected 14 of the 93 genes in this gene ontology. Terms ranked by FC.

To verify the transcriptomics findings, we used qPCR to analyze the effect of *HSPA5* silencing on mRNA expression levels of selected genes in a larger number of specimens. We chose *AP2A1*, which was the most upregulated gene after *HSPA5* silencing, *HSP90B1* as a HSP, *TNFRSF*9 as an example of an inflammatory response pathway component, and *CXCL8* and *CCL2* which were the most affected genes of the IL-17 pathway (Supplementary Tables S2 and S3, Tables 3 and 5). qPCR verified that *HSPA5* silencing upregulated mRNA expression of *AP2A1* and *HSP90B1* and downregulated mRNA expression of *TNFRSF9*, *CXCL8* and *CCL2* (Supplementary Fig. S4, Table S5). These results confirm that *HSPA5* silencing affects the expression of immune response–modifying genes in a placental cell culture model.

## Discussion

HSPA5, also known as 78-kDa glucose-regulated protein (GRP78) and immunoglobulin heavy chain-binding protein (BiP), is a chaperone and a member of the HSP70 family.^44,50,51^ HSPA5 concentrations decrease toward the end of pregnancy in the placenta.^52,53^ Elevated HSPA5 protein expression is observed in preeclamptic placentas^54^ and in preterm placentas compared to term placentas.^55^ Our recent proteomic results showed that HSPA5 is upregulated in the placentas from SPTBs compared to STBs and medically-indicated PTBs.^37^ In this study, we were able to validate upregulation of placental HSPA5 in SPTB vs STB in western blot experiments. Consequently, we studied whether the upregulation was seen at the mRNA level. *HSPA5* mRNA expression levels were shown previously to be elevated in placentas with preeclampsia.^56^ Moreover, *HSPA5* mRNA expression was higher in fetal membranes from SPTBs with ongoing infection.^57^ In our current study, *HSPA5* mRNA expression as measured by qPCR was not increased in SPTB placentas. Thus, we propose that HSPA5 is regulated also post-translationally in the placenta. Mammalian HSPA5 can be post-translationally modified by phosphorylation and ADP ribosylation.^41,44,51^ Post-translational modifications of HSPA5 may regulate the polypeptide binding activity of HSPA5, as well as HSPA5 synthesis.^41^

HSPA5 has two conserved major domains, the NBD and the SBD.^15,25,41,44^ The NBD facilitates ATPase activity and substrate binding ability, whereas the SBD binds substrates such as polypeptides.^15,44^ Together, the major domains regulate both the affinity and duration of substrate binding.^41^ It has been proposed that binding of ATP to the NBD causes the helical lid to open,^58^ while hydrolysis of ATP to ADP causes the lid to close,^44^ which encapsulates the polypeptide in the binding pocket.^41,44^ Recently, we identified a variant of *HSPA5* from WES data^12,37^ that may disrupt the function of the protein. The variant, rs56136100 (E557G), changes glutamic acid into glycine. This change from an acidic to a hydrophobic amino acid may alter the physiochemical properties of the protein.^12^ We illustrated the crystal structure of HSPA5 to investigate the site of the amino acid change. According to the structure, the amino acid in question is in the SBD, at the alpha helical lid. However, the location of the amino acid change does not seem to affect the function of the lid.

Members of the HSP70 family interact with their co-chaperones, such as HSP40s (also known as J-proteins).^15,41,43^ J-proteins may be in specific locations, such as the endoplasmic reticulum (ER)^59^ or mitochondria.^60^ Some J-proteins may bring polypeptide substrates to HSP70s^43,51,60^ and stimulate chaperone activity.^41,44,61^ Interactions between J-protein and HSP70 change the conformation of HSP70, which allows the peptide-binding pocket to close.^60^ J-protein interacts with both the NBD and the SBD.^62^ Since the amino acid change (E557G) is in the SBD of HSPA5, it could affect the interaction between HSPA5 and its co-chaperone. However, this is speculative and should be studied experimentally.

HSPA5 has been observed in trophoblast of fetal membranes from elective term deliveries^57^ and in first trimester syncytio- and cytotrophoblast.^63^ Our immunohistochemical staining revealed HSPA5 in syncytio- and cytotrophoblast cells, as well as in decidual trophoblast within the basal plate of placenta. We did not observe detectable quantitative or qualitative differences in HSPA5 staining between SPTB and STB placentas, indicating that HSPA5 is normally present in trophoblast during the second and third trimesters of pregnancy. Additionally, we observed by immuno-EM that HSPA5 is mainly an intracellular protein. Others have reported HSPA5 in the ER, mitochondria-associated ER membrane, and cell surface.^44,64^ Translocation of HSPA5 to the cell surface is mostly dependent on ER stress.^44^ In the immuno-EM, HSPA5 was mainly in the cytoplasm, and we did not observe any cell organelle specific concentration in SPTB and STB placentas.

ER stress is generally induced by accumulation of unfolded or misfolded proteins within the ER,^44,65^ and HSPA5 participates in the ER stress response.^41,50^ In addition to the ER stress response, HSPA5 may be involved in placentation,^66^ placental trophoblast differentiation,^64^ and trophoblast invasion.^63^ Inappropriate upregulation of ER stress factors may lead to SPTB, especially during ongoing infection.^57^ ER stress has also been observed in other pathophysiological conditions of pregnancy, such as preeclampsia and intrauterine growth restriction.^66^ Labor-associated inflammation could promote ER stress at the fetomaternal interface, creating a positive feedback system.^57^ In our siRNA-induced gene silencing of *HSPA5* in human trophoblast–derived cell culture, the genes most affected were those associated with the inflammatory response. The majority of genes in this pathway were downregulated when *HSPA5* was silenced. The affected genes included proinflammatory chemokines, such as chemoattractants *CCL2* and *CXCL8*/IL-8. CCL2 recruits proinflammatory macrophages during the peri-implantation period,^67^ while IL-8 stimulates migration of several immune cells.^48^ Increased levels of both CCL2 and IL-8 have been observed in the decidua during normal parturition.^57,68^ Accumulation of decidual macrophages has been observed in both spontaneous term and preterm labors, which suggests local production of monocyte chemoattractants in the decidua.^69^ A specific HSP expression profile that includes HSPA5 may be involved in differentiation towards proinflammatory macrophages.^70^ Polarization towards proinflammatory macrophages has been observed in spontaneous term and preterm labor.^71^ These results suggest that HSPA5 may participate in regulation of the immunological state of the placenta.

In conclusion, our results, together with prior knowledge of the mechanisms related to SPTB, suggest that HSPA5 may be an important regulator of the inflammatory state of the placenta. Members of the HSP70 family have been previously associated with SPTB.^12,13^ We propose that the expression pattern of specific HSPs in SPTB involves changes in the inflammatory state at the fetomaternal interface. In the future, a preterm birth model using *HSPA5* transgenic mice may provide stronger validation to our proposal. Based on the present findings, we propose that aberrant expression of placental HSPA5 may predispose to SPTB.

## Supplementary information

Supplemental_file

Supplemental_TablesS1-S3

## Data Availability

RNA sequencing data from the *HSPA5*-silenced HTR8/SVneo cells were deposited in NCBI’s Gene Expression Omnibus database^72^ and are accessible at GEO Series accession number GSE164973. All other relevant data generated or analysed during this study are available within the manuscript and its supplementary information files.
