# Supplementary material for: Elevated human placental heat shock protein 5 is associated with spontaneous preterm birth
Source: Pediatr Res. 2023 Feb 14;94(2):520–9. doi: 10.1038/s41390-023-02501-9 (PMC9926443; doi:10.1038/s41390-023-02501-9)
Supplement: Supplementary file 1 — Supplemental_file [file 41390_2023_2501_MOESM1_ESM.pdf]

Supplemental Table S4. GO Gene Ontology Cellular Component (GO-CC) search of genes affected by *HSPA5*-silencing. *HSPA5* silenced in HTR8/SVneo commercial cell line by siRNA. Transcriptome of these cells compared with transcriptome of cells treated with negative siRNA. GO-CC terms ranked based on *p* value. Threshold of *p* value was <0.05.

Term	Count	%	*p* value[a]	Benjamini
Extracellular region	27	29.3	5.30E-07	9.30E-05
Endoplasmic reticulum lumen	10	10.9	7.10E-06	6.20E-04
Extracellular space	22	23.9	4.70E-05	2.80E-03
Melanosome	5	5.4	1.00E-03	4.50E-02
Extracellular exosome	20	21.7	2.50E-03	8.70E-02
Macromolecular complex	10	10.9	3.00E-03	8.70E-02
Cytosol	36	39.1	4.40E-03	1.00E-01
Membrane raft	6	6.5	4.60E-03	1.00E-01
Cell surface	9	9.8	5.20E-03	1.00E-01
Lysosome	6	6.5	9.70E-03	1.70E-01
Endoplasmic reticulum membrane	11	12	1.20E-02	2.00E-01
Early endosome	5	5.4	3.40E-02	4.80E-01
Clathrin-coated endocytic vesicle membrane	3	3.3	3.70E-02	4.80E-01
Lateral plasma membrane	3	3.3	4.20E-02	4.80E-01
Perinuclear region of cytoplasm	8	8.7	4.40E-02	4.80E-01
Endoplasmic reticulum chaperone complex	2	2.2	4.60E-02	4.80E-01
Endosome	5	5.4	4.70E-02	4.80E-01

[a]Gene ontologies with $p<0.05$ are shown.

Supplemental Table S5. Comparison of RNA sequencing and qPCR of genes affected by *HSPA5* knockdown.

Gene ID	Description	RNA seq: *HSPA5* knockdown		qPCR: *HSPA5* knockdown	
		p value[a]	FC[b]	*p* value[c]	FC[b]
HSPA5	Heat Shock Protein Family A (Hsp70) Member 5	0.00012	-1.8	0.004	-2.1
AP2A1	Adaptor Related Protein Complex 2 Subunit Alpha 1	0.0001	2.0	0.004	1.4
HSP90B1	Heat Shock Protein 90 Beta Family Member 1	0.001	1.9	0.002	1.7
TNFRSF9	TNF Receptor Superfamily Member 9	0.00003	-2.2	0.0002	-3.6
CCL2	C-C motif chemokine ligand 2	0.00011	-1.9	0.002	-2.4
CXCL8	C-X-C motif chemokine ligand 8	0.00020	-1.9	0.002	-2.4

[a]*t*-test *p* value for comparisons between sample groups (*HSPA5*-silenced and negative-control cells).

[b]Expression ratio (FC) between compared sample groups. Comparison between *HSPA5*-silenced cells and negative control cells.

[c]Mann–Whitney *U* test *p* value.

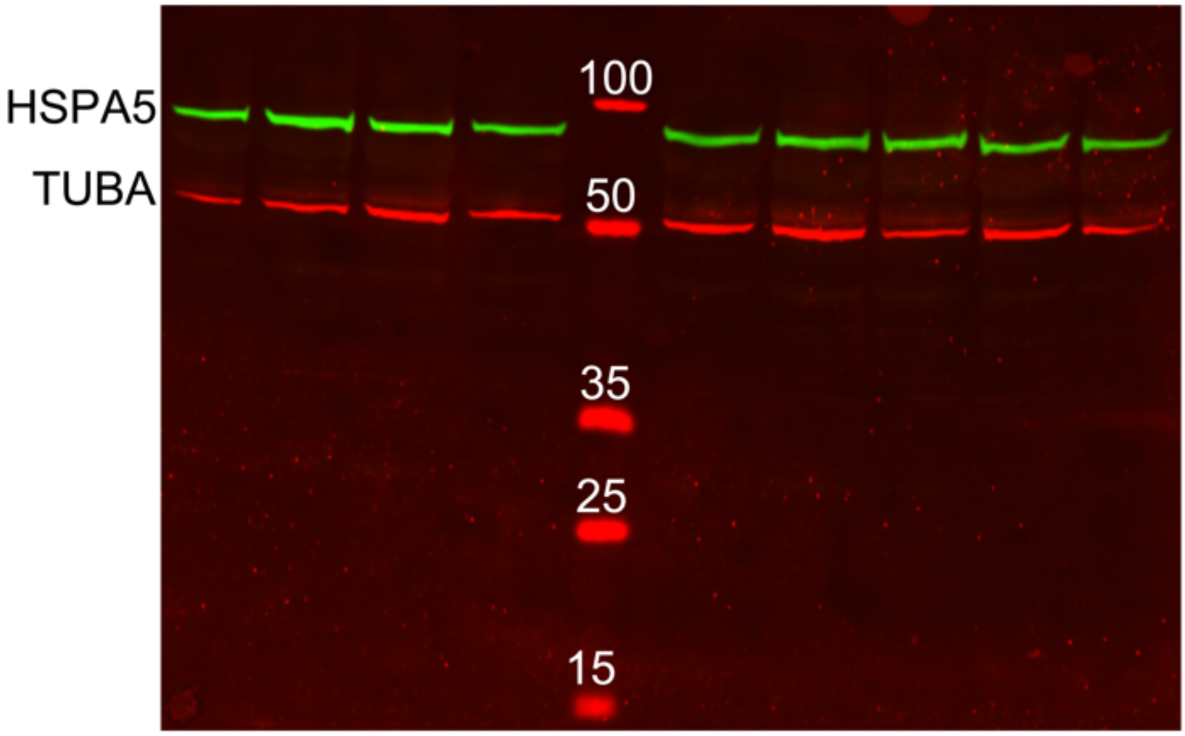

Supplemental Fig. S1. Densitometric evaluation of western blots of HSPA5 (green) and tubulin α-1B (TUBA, red). In this representative western blot, human placental samples from basal plate of STB (*n* = 8) are shown; sample on the right side of protein ladder is the run calibrator. Sizes (kDa) of protein ladder are shown.

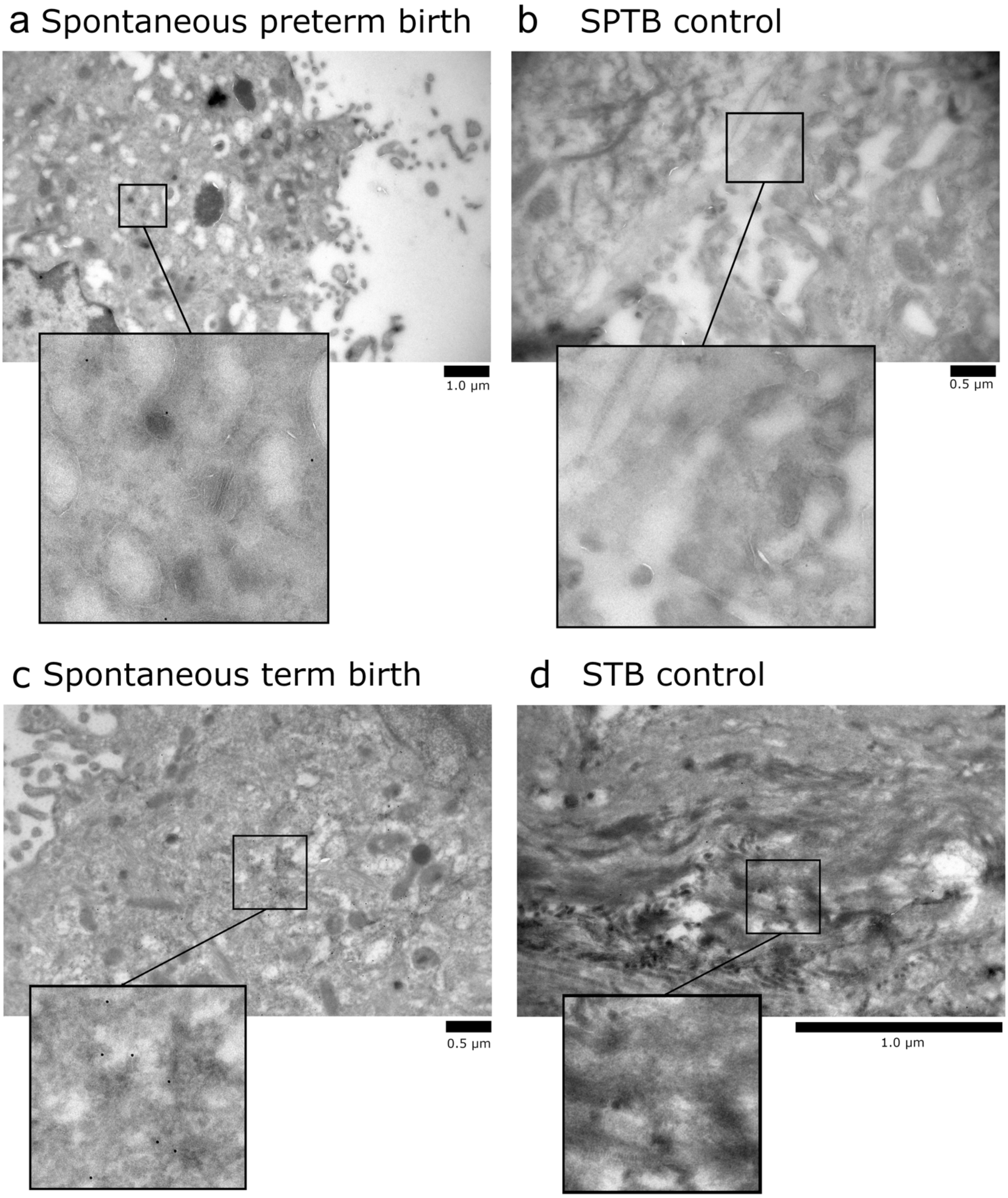

Supplemental Fig. S2. Immunoelectron microscopy of HSPA5 in placenta from spontaneous preterm birth (SPTB) and spontaneous term birth (STB). Samples are from the basal plate. Bound anti-HSPA5 antibodies labeled with protein A–conjugated gold particles. Immunostaining for

HSPA5 in spontaneous preterm birth (a) and spontaneous term birth (c) placenta. In the figures, a part of syncytiotrophoblast and microvilli is shown. Scale bar: 1.0 μm (a) and 0.5 μm (c). For negative controls (b) and (d), the primary antibody was replaced with PBS. Extracellular matrix was chosen as a control because it has a tendency to non-specific binding of protein A–conjugated gold particles. Scale bar: 0.5 μm (b) and 1.0 μm (d).

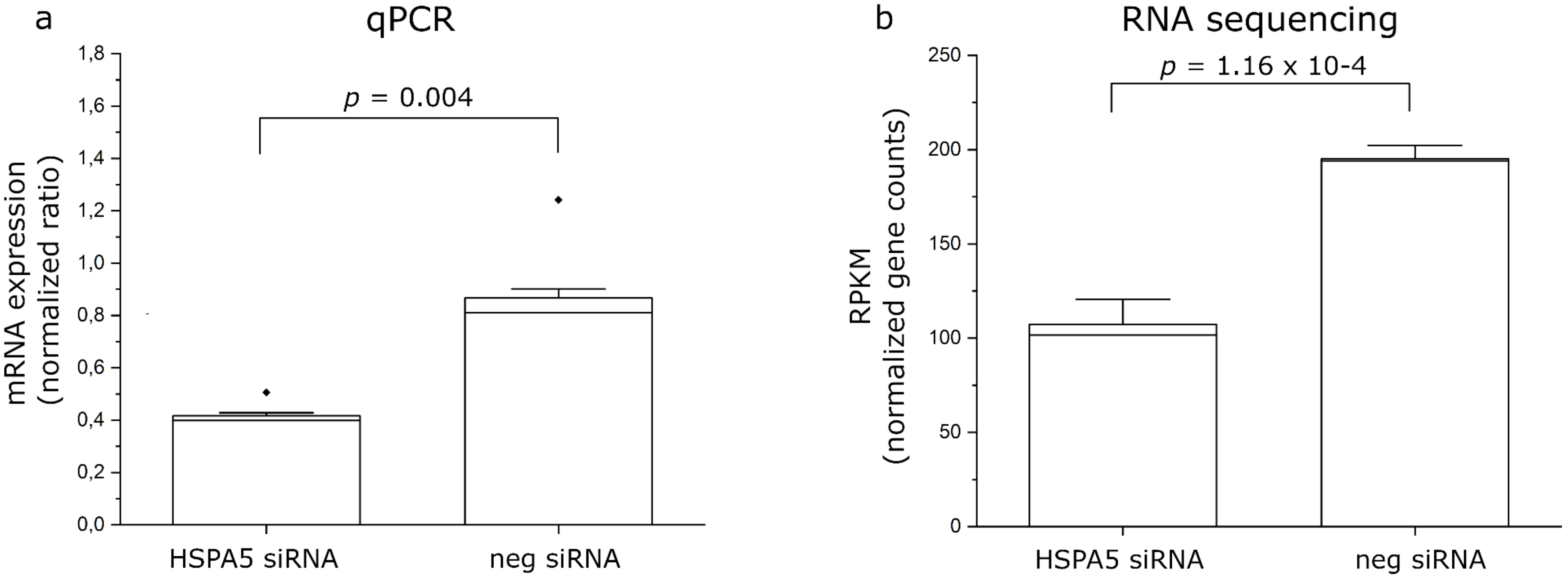

Supplemental Fig. S3. mRNA expression levels after gene knockdown of *HSPA5*. HSPA5 was post-transcriptionally silenced with siRNA in the human placental trophoblast continuous cell line HTR8/SVneo. mRNA levels of cells in which *HSPA5* was silenced compared with mRNA levels from negative siRNA–treated control cells. Expression levels of genes first determined by qPCR (levels normalized against housekeeping gene *CYC1* mRNA levels). According to qPCR, silencing percentage of *HSPA5* was 54% (a). Gene expression levels also determined with high-throughput RNA sequencing. Reads per kilobase of exon per million reads mapped (RPKM) is a normalized gene count value determined in transcriptomic analysis. According to transcriptomics data, silencing percentage was 45% (b). Columns represent median, mean, and standard deviation values.

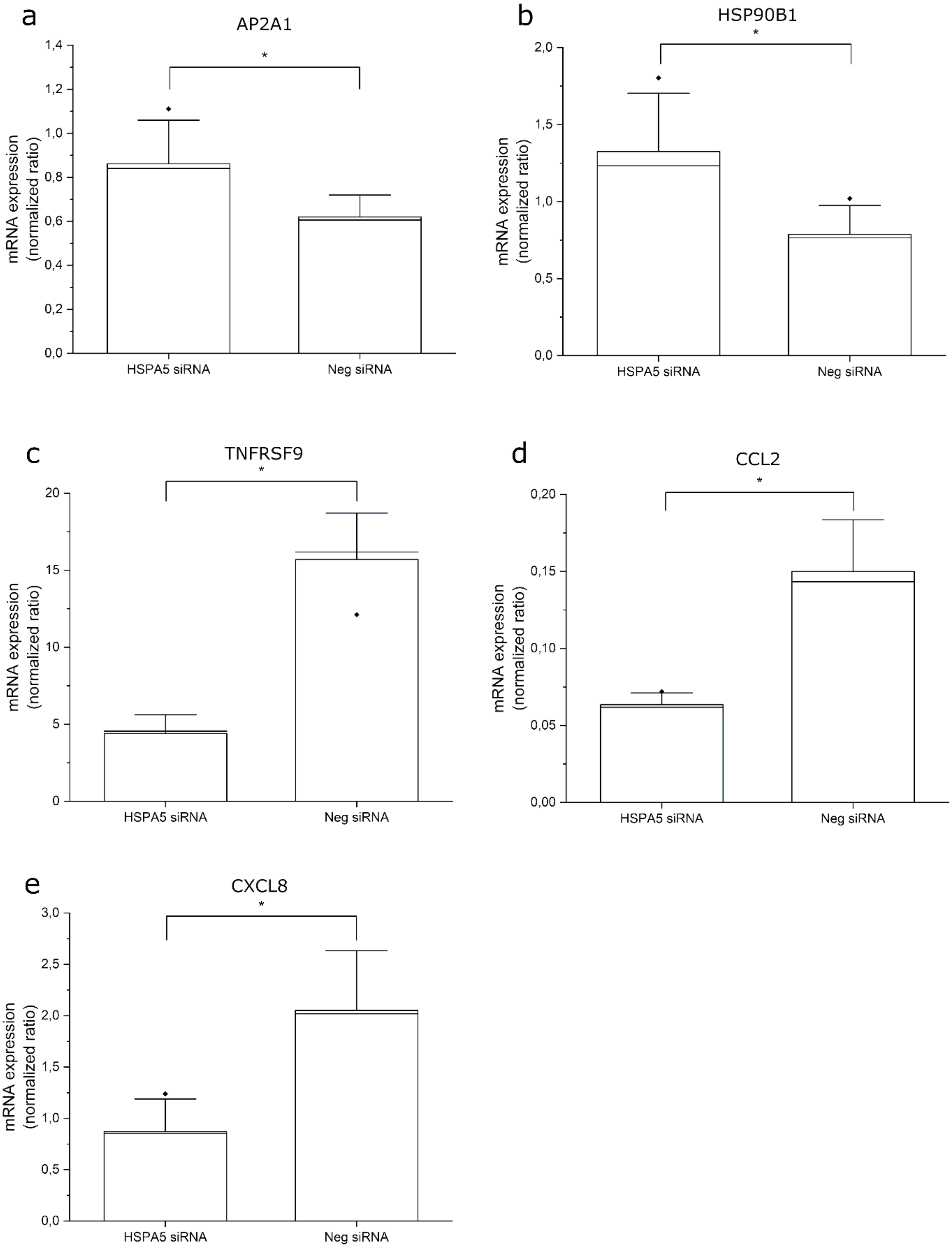

Supplemental Fig. S4. mRNA expression levels after gene knockdown of *HSPA5* assessed by qPCR. RNA sequencing results verified for *AP2A1* (a), *HSP90B1* (b), *TNFRSF9* (c), *CCL2* (d) and *CXCL8* (e). mRNA levels normalized against the housekeeping gene *CYC1*. Statistical analysis performed with Mann–Whitney *U* test. Asterisk represents *p* value of <0.05. Columns represent median, mean, and standard deviation values.
